# Supplementary material for: Human internal exposures to alternariol and its monomethyl ether are predicted below thresholds of in vitro toxicity by physiologically based kinetic modeling
Source: Arch Toxicol. 2026 Jun 27;100(11):5009–25. doi: 10.1007/s00204-026-04485-x (PMC13694328; doi:10.1007/s00204-026-04485-x)
Supplement: Supplementary file 1 [file 204_2026_4485_MOESM1_ESM.zip › Supplementary/S4 Female model for AOH.pdf]

Model S4: Female model for AOH

```
METHOD RK4

STARTTIME = 0
STOPTIME = 10
DT = 0.00002

{Type Equations Here.}
{Top model}
 {Reservoirs}

d/dt (ASTAOH) = - transSTtoSI + oraldose
INIT ASTAOH = 0
LIMIT ASTAOH >= 0
d/dt (ASIAOH) = -transSItoSIG - transSItoLI  +  transSTtoSI
INIT ASIAOH = 0
LIMIT ASIAOH >= 0
d/dt (ALIAOH) =  +  transSItoLI - fecalex - transLItoL
INIT ALIAOH = 0
LIMIT ALIAOH >= 0
d/dt (AGSIAOH) =  + transSItoSIG  -venGS
INIT AGSIAOH = 0
LIMIT AGSIAOH >= 0
d/dt (FECAOH) = + fecalex
INIT FECAOH = 0
LIMIT FECAOH >= 0
d/dt (ALAOH) = - venL + artL + venP + venGS  + transLItoL - Phase1metab - AOHglucur
INIT ALAOH = 0
LIMIT ALAOH >= 0
d/dt (ABAOH) = + venL + venS + venR + venF + venK + venM  - artL - artP - artS - artR - artF - artK - artM
INIT ABAOH = 0
LIMIT ABAOH >= 0
d/dt (APAOH) = + artP - venP
INIT APAOH = 0
LIMIT APAOH >= 0
d/dt (ASAOH) = + artS - venS
INIT ASAOH = 0
LIMIT ASAOH >= 0
d/dt (ARAOH) = + artR - venR
INIT ARAOH = 0
LIMIT ARAOH >= 0
d/dt (AFAOH) = + artF - venF
INIT AFAOH = 0
LIMIT AFAOH >= 0
d/dt (AKAOH) = + artK - venK - urineAOH
INIT AKAOH = 0
LIMIT AKAOH >= 0
d/dt (AUCAOHUr) = + urineAOH
INIT AUCAOHUr = 0
d/dt (AMAOH) = + artM - venM
INIT AMAOH = 0
LIMIT AMAOH >= 0
d/dt (Start) = - oraldose
INIT Start = AODOSEAOH
LIMIT Start >=0
d/dt (exAOHmetab) = + Phase1metab
INIT exAOHmetab = 0
LIMIT exAOHmetab >= 0
d/dt (exAOHglucur) = + AOHglucur
INIT exAOHglucur = 0
LIMIT exAOHglucur >= 0
d/dt (AUAOH) = + artU - venU
INIT AUAOH = 0
```

```
 LIMIT AUAOH >= 0

{Flows}

; arterial (art) and venous (ven) blood flows :

venL = QL*CVLAOH
artL = (QL-QP)*CBAOH
venP = QP*CPAOH
artP = QP*CBAOH
venS = QS*CVSAOH
artS = QS*CBAOH
venR = QR*CVRAOH
artR = QR*CBAOH
venF = QF*CVFAOH
artF = QF*CBAOH
venM = QM*CVMAOH
artM = QM*CBAOH
venK = QK*CVKAOH
artK = QK*CBAOH
venGS = QSI*CVGSIAOH
artGS = QSI*CBAOH
venU = QU*CVUAOH
artU = QU*CBAOH

; excretion :

urineAOH = GFR*CVKAOH*60/1000  ;  (L/h)
fecalex = Kli*ALIAOH

; gastrointestinal tract :

transSTtoSI = kelST * ASTAOH                    ; from stomach lumen to small intestine lumen
transSItoSIG = KaAOH*CSIAOH                     ; from small intestine lumen to gut small intestine tissue
transSItoLI = ASIAOH*Ksi                        ; from small intestine lumen to large intestine lumen
transLItoL = KbAOH*CLIAOH                       ; from large intestine lumen to liver tissue

oraldose = PULSE(AODOSEAOH, 0, 24)

;Liver metabolism :
Phase1metab = VmaxLAOH * CVLAOH/(KmLAOH + CVLAOH)          ; MichaelisMenten
AOHglucur = VmaxLAOHgluc * CVLAOH/(KmLAOHgluc + CVLAOH)  ; MichaelisMenten

{Globals}
; AOH female model
;=================================================================
; Physiological parameters ;=================================================================
;bodyweight
BW = 60               ;Kg         ; avg. BW in validation study (ICRP)
;-------------------------------------------------------------------------------------------------------------------
; relative tissue volumes
; fraction of BW
VLc = 0.0298                     ; fraction of liver tissue (ICRP)
VPc = 0.0064                     ; fraction portal vein perfused tissue (stomach, spleen, pancreas) tissue (ICRP)
VRc =0.0199                      ; fraction of rapidly perfused tis. (Q/V >>1, heart + lungs) tissue (ICRP)
VSc = 0.1748                     ; fraction of slowly perfused tissue (bone, skin) tissue (ICRP)
VFc = 0.3055                     ; fraction of fat tissue (adipose) tissue (ICRP)
VMc = 0.2985                     ; fraction of muscle tissue tissue (ICRP)
VKc = 0.0059                     ; fraction of kidneys tissue (ICRP)
VSIc = 0.0125                    ; fraction of small intestine tissue (ICRP)
VLIc = 0.0074                    ; fraction of large intestine tissue (ICRP)
VBc = 0.065                      ; fraction of blood (ICRP)
VUc= 0.0016                      ;fraction of uterus tissue (ICRP)
;-------------------------------------------------------------------------------------------------------------------
```

```
; calculated tissue volumes
VL = VLc*BW       ;L or Kg                          ; volume of liver tissue (calculated)
VP = VPc*BW       ;L or kg                          ; volume of portal vein perfused tissue
VR = VRc*BW        ;L or Kg                         ; volume of rapidly perfused tissue (calculated)
VS = VSc*BW       ;L or Kg                          ; volume of slowly perfused tissue (calculated)
VF = VFc*BW       ;L or Kg                          ; volume of fat tissue (calculated)
VM = VMc*BW       ;L or Kg                          ; volume of muscle tissue (calculated)
VK = VKc*BW       ;L or Kg                          ; volume of kidney (calculated)
VB = VBc*BW       ;L or Kg                          ; volume of blood (Brown et al, calculated)
VSI = VSIc*BW     ;L or Kg                          ; volume of small intestine tissue (calculated)
VLI = VLIc*BW     ;L or Kg                          ; volume of large intestine tissue  (calculated)
VU = Vuc*BW       ;L or Kg                          ; volume of uterus tissue (calculated)
VSIL = 4.13       ;L or Kg                          ; volume of small intestine lumen (ICRP, calculated)
VLIL = 2.16       ;L or Kg                          ; volume of large intestine lumen (ICRP, calculated)
;------------------------------------------------------------------------------------------------------------------
;surface areas

areaSI = 2709.066;dm2        ;surface area of SI lumen (Helander et al)
areaLI = 98.018;dm2          ;surface area of LI lumen (Helander et al)
;------------------------------------------------------------------------------------------------------------------
;Blood flow rates,
QCc = 5.9           ; (L/min), cardiac output (ICRP)
QPc = 0.05          ; fraction of blood flow to P (spleen, stom., pancreas) (ICRP)
QLc = 0.065         ; fraction of blood flow to liver in total (ICRP)
QRc = 1.05          ; fraction of blood flow to rapidly perfused tissue (heart+lungs)        (ICRP)
QSc = 0.1           ; fraction of blood flow to slowly perfused tissue (skin+Bones)          (ICRP)
QFc = 0.085         ; fraction of blood flow to fat  (ICRP)
QMc =0.12           ; fraction of blood flow to muscle (ICRP)
QKc =0.17           ; fraction of blood flow to kidneys (ICRP)
QSIc =0.11          ; fraction of blood flow to small intestine (ICRP)
QUc= 0.004          ;fraction of blood to uterus (ICRP)

QC = QCc*60                  ; (L/h)              ; cardiac output (calculated)
QP = QPc*QC                  ;L or Kg  ; blood flow to portal vein perfused tissue (calculated)
QL = QLc*QC                  ;L or Kg  ; blood flow to liver total (calculated)
QR = QRc*QC                  ;L or Kg  ; blood flow to rapidly perfused tissue (calculated)
QS = QSc*QC                  ;L or Kg  ; blood flow to slowly perfused tissue (calculated)
QF = QFc*QC                  ;L or Kg  ; blood flow to fat tissue (calculated)
QM = QMc*QC                  ;L or Kg  ; blood flow to muscle tissue (calculated)
QK = QKc*QC                  ;L or Kg  ; blood flow to kidney (calculated)
QSI=QSIc*QC                  ;L or Kg  ; blood flow to small intestine tissue (calculated)
QU = QUc*QC                  ;L or Kg  ; blood flow to uterus tissue (calculated)
;------------------------------------------------------------------------------------------------------------------
; glomerular filtration rate
GFR = 125           ;mL/min                ; GFR scaled to body weight (Levey et al.)
;=================================================================
; Physicochemical parameters
;=================================================================
; molecular weights (g/mol)
MWAOH = 258.23 ; parent compound
; pKa1
pKa1AOH =7.63 ; parent compound Chemaxon playground (v1.6.2)
; pKa2
pKaAOH =8.44 ; parent compound Chemaxon playground (v1.6.2)
; logP
logPAOH = 3.18 ; parent compound Chemaxon playground (v1.6.2)
; logD (parent compound)
logDapical = 3.17 ;Chemaxon playground (v1.6.2)
logDbasal = 2.96 ; Chemaxon playground (v1.6.2)

; partition coefficients                    ;calculated by qivivetools.wu.nl (Berezhkovskiy algorithm)
; Alternariol in main model
PLAOH = 3.21                                ; liver/blood partition coefficient
PRAOH = 0.894                ; rapidly perfused tissue/blood partition coefficient
```

```
PSAOH = 4.635               ; slowly perfused tissue/blood partition coefficient
PFAOH = 7.3                 ; fat/blood partition coefficient
PMAOH = 2.09                ; muscle/blood partition coefficient
PKAOH = 2.07                ; kidney/blood partition coefficient
FUAOH = 0.06                ; faction unbound plasma according to Lobell&Sivarajah
PSIAOH =3.97                ; gut/blood partition coefficient
;-----------------------------------------------------------------------------------------------------------
; absorption/transfer rates
logPappAOH = -5.09                  ;log(cm/s)          ;log permeability calculated from Caco2 data (Burkhardt et al)
PeffAOH = (3600/10)*(10^(0.6836*LogPappAOH- 0.5579))        ;dm/h     ;scaled according toSun et al.


KaAOH = PeffAOH*areaSI              ;L/h ;transfer rate of AOH from SI lumen to gut
KbAOH = PeffAOH*areaLI              ;L/h ;transfer rate of AOH from LI lumen to gut

; gastric emptying (kelst)
GEstc = 11.8                        ; (min) half time of meal in stomach
GEst = GEstc /60            ; (h)
LNhalf= -0.69314718                 ; LN(1/2)
kelST =  -LNhalf/ GEst      ; (h-1) constant of gastric excretion              ; from Ct=C0*e^(-kel*t)

tpassSI = 4                 ;h          ;passing time through small intestine (ICRP)
Ksi = 1/tpassSI             ;/h                 ;SI to LI transfer constant
tpassLI = 48                ;h                  ;passing time through large intestine (ICRP)
Kli = 1/tpassLI             ;/h                 ;LI to excretion transfer constant
;==================================================================
; Kinetic parameters
;==================================================================
;metabolism of rat liver
; scaling factors
VLS9 = 107.3      ;mg S9 protein/gram liver             ; liver S9 protein yield (Wang et al. 2019)
VLMP = 32         ;mg protein/gram liver                ; liver microsomal protein yield (Barter et al. 2007)

;Phase 1 metabolism
KmLAOH = 131                ;µmol/L  ;Michaelis constant of hepatic phase I metabolism          (Borsos et al)
VmaxLAOHc = 0.00337                 ;µmol/mg/min   microsomal protein (Borsos et al)
VmaxLAOH = VmaxLAOHc*VLMP*VL*60*10^3          ;µmol/h

;Phase 2 glucuronidation
KmLAOHgluc = 2.7                            ;µmol/L             ;Michaelis constant of hepatic glucuronidation (in vitro)
VmaxLAOHglucc =0.0069               ;µmol/(mg*min) (in vitro)
VmaxLAOHgluc = VmaxLAOHglucc*VLS9*VL*60*10^3            ;µmol/h
;==================================================================
; Run settings
;===================================================================
;-----------------------------------------------------------------------------------------------------------
; oral dose
ODOSEAOH = 0.0022*BW  ;µg                   ;oral dose alternariol, variable
AODOSEAOH = ODOSEAOH/MWAOH          ;umol               ;oral dose AOH in umol
;===================================================================
; Main model calculations/dynamics:
;===================================================================
;-----------------------------------------------------------------------------------------------------------
;ASIAOH: amount of AOH in small intestinal lumen compartment  ;µmol
CSIAOH=ASIAOH/VSIL                                            ;umol/L
;-----------------------------------------------------------------------------------------------------------
;large intestine compartment
;ALIAOH: amount of AOH in large intestine lumen                ;µmol
CLIAOH = ALIAOH/VLIL                                           ;umol/L

;--------------------------------------------------------------------------------------------------------

;gut small intestine tissue compartment

;AGSIAOH: amount of AOH in small intestine tissue               ;µmol
```

```
CGSIAOH = AGSIAOH/VSI                                   ;µmol/L

CVGSIAOH = CGSIAOH/PSIAOH
;-----------------------------------------------------------------------------------------------------------------
; liver compartment
; ALAOH: amount of AOH in liver ;µmol
CLAOH = ALAOH/VL                 ;umol/L
CVLAOH = CLAOH/PLAOH             ;partition with liver tissue
;-----------------------------------------------------------------------------------------------------------------
; portal vein perfused tissue compartment
; APAOH = amount of AOH in spleen tissue ;µmol
CPAOH = APAOH/VP                 ;umol/L
;----------------------------------------------------------------------------------------------------------------
; fat (adipose tissue) compartment
CFAOH = AFAOH/VF                 ;umol/L
CVFAOH = CFAOH/PFAOH             ;partition with adipose tissue
;-----------------------------------------------------------------------------------------------------------------
; muscle compartment
; AMAOH = amount of AOH in muscle tissue    ;µmol
CMAOH = AMAOH/VM                 ;umol/L
CVMAOH = CMAOH/PMAOH             ;partition with muscle tissue
;---------------------------------------------------------------------------------------------------------------
; rapidly perfused tissue
CRAOH = ARAOH/VR                 ;umol/L
CVRAOH = CRAOH/PRAOH             ;partition with muscle tissue
;-----------------------------------------------------------------------------------------------------------------
; slowly perfused tissue
CSAOH = ASAOH/VS                 ;umol/L
CVSAOH = CSAOH/PSAOH             ;partition with slowly perfused tissue
;-----------------------------------------------------------------------------------------------------------------
; kidney
CKAOH = AKAOH/VK                 ;umol/L
CVKAOH = CKAOH/PKAOH             ;partition with kidney tissue
;----------------------------------------------------------------------------------------------------------------
; blood compartment
CBAOH=ABAOH/VB                   ;umol/L
CVBAOH = CBAOH*FUAOH                        ;free concentration in plasma
CBAOHug = CBAOH*MWAOH            ;µg/L = ng/mL                ;concentration in blood in ng/ml
;-----------------------------------------------------------------------------------------------------------------
; uterus compartment
CUAOH=AUAOH/VU                   ;umol/L
CVUAOH = CUAOH*PMAOH                        ;free concentration in plasma
;-----------------------------------------------------------------------------------------------------------------
;=====================================================================
; Main model: mass balance calculation
;======================================================================
Total = AODOSEAOH
Calculated = ASTAOH + ASIAOH  + ALIAOH + AGSIAOH  + ALAOH + ABAOH + AKAOH + ARAOH + ASAOH +
AFAOH + APAOH + AMAOH + AUCAOHUr + FECAOH  +exAOHmetab +exAOHglucur + AUAOH

ERROR=((Total-Calculated)/(Total+1E-30))*100
MASSBAL=Total-Calculated + 1

{End Globals}
```
