# Supplementary material for: Human internal exposures to alternariol and its monomethyl ether are predicted below thresholds of in vitro toxicity by physiologically based kinetic modeling
Source: Arch Toxicol. 2026 Jun 27;100(11):5009–25. doi: 10.1007/s00204-026-04485-x (PMC13694328; doi:10.1007/s00204-026-04485-x)
Supplement: Supplementary file 1 [file 204_2026_4485_MOESM1_ESM.zip › Supplementary/S7 Male model for AME.pdf]

Model S7: Male model for AME

```
METHOD RK4

STARTTIME = 0
STOPTIME = 10
DT = 0.00002

{Type Equations Here.}
{Top model}
{Reservoirs}

d/dt (ASTAME) = - transSTtoSI + oraldose
INIT ASTAME = 0
LIMIT ASTAME >= 0
d/dt (ASIAME) = -transSItoSIG - transSItoLI  +  transSTtoSI
INIT ASIAME = 0
LIMIT ASIAME >= 0
d/dt (ALIAME) =  +  transSItoLI - fecalex - transLItoL
INIT ALIAME = 0
LIMIT ALIAME >= 0
d/dt (AGSIAME) =  + transSItoSIG  -venGS
INIT AGSIAME = 0
LIMIT AGSIAME >= 0
d/dt (FECAME) = + fecalex
INIT FECAME = 0
LIMIT FECAME >= 0
d/dt (ALAME) = - venL + artL + venP + venGS  + transLItoL - Phase1metab - AMEglucur
INIT ALAME = 0
LIMIT ALAME >= 0
d/dt (ABAME) = + venL + venS + venR + venF + venK + venM  - artL - artP - artS - artR -artF - artK - artM
INIT ABAME = 0
LIMIT ABAME >= 0
d/dt (APAME) = + artP - venP
INIT APAME = 0
LIMIT APAME >= 0
d/dt (ASAME) = + artS - venS
INIT ASAME = 0
LIMIT ASAME >= 0
d/dt (ARAME) = + artR - venR
INIT ARAME = 0
LIMIT ARAME >= 0
d/dt (AFAME) = + artF - venF
INIT AFAME = 0
LIMIT AFAME >= 0
d/dt (AKAME) = + artK - venK - urineAME
INIT AKAME = 0
LIMIT AKAME >= 0
d/dt (AUCAMEUr) = + urineAME
INIT AUCAMEUr = 0
d/dt (AMAME) = + artM - venM
INIT AMAME = 0
LIMIT AMAME >= 0
d/dt (Start) = - oraldose
INIT Start = AODOSEAME
LIMIT Start >=0
d/dt (exAMEmetab) = + Phase1metab
INIT exAMEmetab = 0
LIMIT exAMEmetab >= 0
d/dt (exAMEglucur) = + AMEglucur
INIT exAMEglucur = 0
LIMIT exAMEglucur >= 0

{Flows}
```

```
 ; arterial (art) and venous (ven) blood flows :
venL = QL*CVLAME
artL = (QL-QP)*CBAME
venP = QP*CPAME
artP = QP*CBAME
venS = QS*CVSAME
artS = QS*CBAME
venR = QR*CVRAME
artR = QR*CBAME
venF = QF*CVFAME
artF = QF*CBAME
venM = QM*CVMAME
artM = QM*CBAME
venK = QK*CVKAME
artK = QK*CBAME
venGS = QSI*CVGSIAME
artGS = QSI*CBAME

 ; excretion :
urineAME = GFR*CVKAME*60/1000  ;  (L/h)
fecalex = Kli*ALIAME

; gastrointestinal tract :
transSTtoSI = kelST * ASTAME                ; from stomach lumen to small intestine lumen
transSItoSIG = KaAME*CSIAME                 ; from small intestine lumen to gut small intestine tissue
transSItoLI = ASIAME*Ksi                    ; from small intestine lumen to large intestine lumen
transLItoL = KbAME*CLIAME                   ; from large intestine lumen to liver tissue

oraldose = PULSE(AODOSEAME, 0, 24)

;Liver metabolism :
Phase1metab = VmaxLAME * CVLAME/(KmLAME + CVLAME)         ; MichaelisMenten
AMEglucur = VmaxLAMEgluc * CVLAME/(KmLAMEgluc + CVLAME)  ; MichaelisMenten

{Globals}

;AME male model
;==================================================================
; Physiological parameters
;==================================================================
;bodyweight
BW = 73 ;Kg          ; avg. BW in validation study (ICRP)
;-----------------------------------------------------------------------------------------------------------------
; relative tissue volumes
; fraction of BW
VLc = 0.0319                 ; fraction of liver tissue (ICRP)
VPc = 0.0063                 ; fraction portal vein perfused tissue (stomach, spleen, pancreas) tissue (ICRP)
VRc =0.0212                  ; fraction of rapidly perfused tis. (Q/V >>1, heart + lungs) tissue (ICRP)
VSc = 0.1963                 ; fraction of slowly perfused tissue (bone, skin) tissue (ICRP)
VFc = 0.2036                 ; fraction of fat tissue (adipose) tissue (ICRP)
VMc = 0.4074                 ; fraction of muscle tissue tissue (ICRP)
VKc = 0.0057                 ; fraction of kidneys tissue (ICRP)
VSIc = 0.0117                ; fraction of small intestine tissue (ICRP)
VLIc = 0.0067                ; fraction of large intestine tissue (ICRP)
VBc = 0.0726                 ; fraction of blood (ICRP)


;-----------------------------------------------------------------------------------------------------------------
; calculated tissue volumes
VL = VLc*BW            ;L or Kg               ; volume of liver tissue (calculated)
VP = VPc*BW            ;L or kg               ; volume of portal vein perfused tissue
VR = VRc*BW             ;L or Kg              ; volume of rapidly perfused tissue (calculated)
VS = VSc*BW            ;L or Kg               ; volume of slowly perfused tissue (calculated)
VF = VFc*BW            ;L or Kg               ; volume of fat tissue (calculated)
```

```
VM = VMc*BW          ;L or Kg          ; volume of muscle tissue (calculated)
VK = VKc*BW          ;L or Kg          ; volume of kidney (calculated)
VB = VBc*BW          ;L or Kg          ; volume of blood (Brown et al, calculated)
VSI = VSIc*BW        ;L or Kg          ; volume of small intestine tissue (calculated)
VLI = VLIc*BW        ;L or Kg          ; volume of large intestine tissue  (calculated)
VSIL = 4.45          ;L or Kg          ; volume of small intestine lumen (ICPR, calculated)
VLIL = 2.38          ;L or Kg          ; volume of large intestine lumen (ICPR, calculated);-----------------
-------------------------------------------------------------------------------------------------
;surface areas
areaSI = 2917.5      ;dm2        ;surface area of SI lumen (Helander et al.)
areaLI = 107.8       ;dm2        ;surface area of LI lumen (Helander et al.)
;----------------------------------------------------------------------------------------------------------------
;Blood flow rates,
QCc = 6.5            ; (L/min), cardiac output (ICRP)
QPc = 0.05           ; fraction of blood flow to P (spleen, stom., pancreas) (ICRP)
QLc = 0.065          ; fraction of blood flow to liver in total (ICRP)
QRc = 1.04           ; fraction of blood flow to rapidly perfused tissue (heart+lungs)      (ICRP)
QSc = 0.1            ; fraction of blood flow to slowly perfused tissue (skin+Bones)        (ICRP)
QFc = 0.05           ; fraction of blood flow to fat  (ICRP)
QMc =0.17            ; fraction of blood flow to muscle (ICRP)
QKc =0.19            ; fraction of blood flow to kidneys (ICRP)
QSIc =0.1            ; fraction of blood flow to small intestine (ICRP)
QLIc=0.04            ; fraction of blood flow to large intestine (ICRP)

QC = QCc*60          ; (L/h)       ; cardiac output (calculated)
QP = QPc*QC          ;L or Kg      ; blood flow to portal vein perfused tissue (calculated)
QL = QLc*QC          ;L or Kg      ; blood flow to liver total (calculated)
QR = QRc*QC          ;L or Kg      ; blood flow to rapidly perfused tissue (calculated)
QS = QSc*QC          ;L or Kg      ; blood flow to slowly perfused tissue (calculated)
QF = QFc*QC          ;L or Kg      ; blood flow to fat tissue (calculated)
QM = QMc*QC          ;L or Kg      ; blood flow to muscle tissue (calculated)
QK = QKc*QC          ;L or Kg      ; blood flow to kidney (calculated)
QSI=QSIc*QC          ;L or Kg      ; blood flow to small intestine tissue (calculated)
QLI=QLIc*QC          ;L or Kg      ; blood flow to large intestine tissue (calculated)
;----------------------------------------------------------------------------------------------------------------
; glomerular filtration rate
GFR = 125      ;mL/min        ; GFR scaled to body weight (Levey et al.)
;=================================================================
; Physicochemical parameters
;=================================================================
; molecular weights (g/mol)
MWAME = 272.25          ; parent compound
; pKa1
pKa1AME =7.71 ; parent compound Chemaxon playground (v1.6.2)
; logP
logPAME = 3.32 ; parent compound Chemaxon playground (v1.6.2)
; logD (parent compound)
;logDapical = 3.32 Chemaxon playground (v1.6.2)
;logDbasal = 3.1 Chemaxon playground (v1.6.2)

; partition coefficients          ;calculated by qivivetools.wu.nl (Berezhkovskiy algorithm)
; AME in main model
PLAME =3.8           ; liver/blood partition coefficient
PRAME = 0.982        ; rapidly perfused tissue/blood partition coefficient
PSAME = 5.509        ; slowly perfused tissue/blood partition coefficient
PFAME = 7.1          ; fat/blood partition coefficient
PMAME = 2.44         ; muscle/blood partition coefficient
PKAME = 2.41         ; kidney/blood partition coefficient
FUAME = 0.058        ; faction unbound plasma according to Lobell&Sivarajah
PSIAME =4.72         ; gut/blood partition coefficient
;----------------------------------------------------------------------------------------------------------------
; absorption/transfer rates
logPappAME = -5.11          ;log(cm/s)                    ;log permeability estimated
```

```
PeffAME = (3600/10)*(10^(0.6836*LogPappAME- 0.5579))          ;dm/h     ;scaled according to Sun et al.


KaAME = PeffAME*areaSI              ;L/h ;transfer rate of AME from SI lumen to gut
KbAME = PeffAME*areaLI              ;L/h ;transfer rate of AAME from LI lumen to gut

; gastric emptying (kelst)
GEstc = 11.8                        ; (min) half time of meal in mice stomach
GEst = GEstc /60              ; (h)
LNhalf= -0.69314718                 ; LN(1/2)
kelST =  -LNhalf/ GEst        ; (h-1) constant of gastric excretion                 ; from Ct=C0*e^(-kel*t)

tpassSI = 4          ;h        ;passing time through small intestine (ICRP)
Ksi = 1/tpassSI      ;/h       ;SI to LI transfer constant
tpassLI = 36         ;h        ;passing time through large intestine (ICRP)
Kli = 1/tpassLI      ;/h       ;LI to excretion transfer constant
;==================================================================
; Kinetic parameters
;==================================================================
;metabolism of rat liver
; scaling factors
VLS9 = 107.3         ;mg S9 protein/gram liver               ; liver S9 protein yield (Wang et al. 2019)
VLMP = 32            ;mg protein/gram liver                  ; liver microsomal protein yield (Barter et al. 2007)

;Phase 1 metabolism
KmLAME = 29.4                       ;µmol/L             ;Michaelis constant of hepatic phase I metabolism (Borsos et al)
VmaxLAMEc = 0.000772                           ;µmol/mg/min   microsomal protein (Borsos et al)
VmaxLAME = VmaxLAMEc*VLMP*VL*60*10^3           ;µmol/h

;Phase 2 glucuronidation

KmLAMEgluc = 0.97                              ;µmol/L             ;Michaelis constant of hepatic glucuronidation (in vitro)
VmaxLAMEglucc =0.0105               ;µmol/(mg*min) (in vitro)
VmaxLAMEgluc = VmaxLAMEglucc*VLS9*VL*60*10^3             ;µmol/h
;==================================================================
; Run settings
;==================================================================
;------------------------------------------------------------------------------------------------------------------
; oral dose
ODOSEAME = 0.0014*BW ;µg                              ;oral dose alternariol, variable
AODOSEAME = ODOSEAME/MWAME          ;umol                 ;oral dose AME in umol
;==================================================================
; Main model calculations/dynamics:
;==================================================================
;------------------------------------------------------------------------------------------------------------------
;ASIAME: amount of AME in small intestinal lumen compartment               ;µmol
CSIAME=ASIAME/VSIL                                                 ;umol/L
;------------------------------------------------------------------------------------------------------------------
;large intestine compartment
;ALIAME: amount of AME in large intestine lumen                               ;µmol
CLIAME = ALIAME/VLIL                                               ;umol/L
;------------------------------------------------------------------------------------------------------------------
;gut small intestine tissue compartment
;AGSIAME: amount of AME in small intestine tissue                             ;µmol
CGSIAME = AGSIAME/VSI                                                         ;µmol/L
CVGSIAME = CGSIAME/PSIAME
;------------------------------------------------------------------------------------------------------------------
; liver compartment
; ALAME: amount of AME in liver ;µmol
CLAME = ALAME/VL                  ;umol/L
CVLAME = CLAME/PLAME                           ;partition with liver tissue
;------------------------------------------------------------------------------------------------------------------
; portal vein perfused tissue compartment
; APAME = amount of AME in spleen tissue ;µmol
```

```
CPAME = APAME/VP                    ;umol/L
;------------------------------------------------------------------------------------------------------------------
; fat (adipose tissue) compartment
CFAME = AFAME/VF                    ;umol/L
CVFAME = CFAME/PFAME                          ;partition with adipose tissue
;------------------------------------------------------------------------------------------------------------------
; muscle compartment
; AMAME = amount of AME in muscle tissue    ;µmol
CMAME = AMAME/VM                    ;umol/L
CVMAME = CMAME/PMAME                          ;partition with muscle tissue
;------------------------------------------------------------------------------------------------------------------
; rapidly perfused tissue
CRAME = ARAME/VR                    ;umol/L
CVRAME = CRAME/PRAME                          ;partition with muscle tissue
;-----------------------------------------------------------------------------------------------------------------
; slowly perfused tissue
CSAME = ASAME/VS                    ;umol/L
CVSAME = CSAME/PSAME                          ;partition with slowly perfused tissue
;------------------------------------------------------------------------------------------------------------------
; kidney
CKAME = AKAME/VK                    ;umol/L
CVKAME = CKAME/PKAME                          ;partition with kidney tissue
;------------------------------------------------------------------------------------------------------------------
; blood compartment
CBAME=ABAME/VB                      ;umol/LCVBAME = CBAME*FUAME                  ;free    concentration    in
plasma
CBAMEug = CBAME*MWAME       ;µg/L = ng/mL                    ;concentration in blood in ng/ml
;------------------------------------------------------------------------------------------------------------------
;=================================================================
; Main model: mass balance calculation
;=================================================================
Total = AODOSEAME
Calculated = ASTAME + ASIAME  + ALIAME + AGSIAME  + ALAME+ ABAME+ AKAME + ARAME + ASAME +
AFAME + APAME + AMAME + AUCAMEUr +FECAME+exAMEmetab + exAMEglucur

ERROR=((Total-Calculated)/(Total+1E-30))*100
MASSBAL=Total-Calculated + 1

{End Globals}
```
